# Approaches to treatment of emerging Shiga toxin-producing *Escherichia coli* infections highlighting the O104:H4 serotype

**DOI:** 10.3389/fcimb.2015.00024

**Published:** 2015-03-18

**Authors:** Elias A. Rahal, Sukayna M. Fadlallah, Farah J. Nassar, Natalie Kazzi, Ghassan M. Matar

**Affiliations:** Department of Experimental Pathology, Immunology and Microbiology, Faculty of Medicine, American University of BeirutBeirut, Lebanon

**Keywords:** Shiga toxin-producing *Escherichia coli*, hemorrhagic colitis, hemolytic uremic syndrome, antimicrobial agents, Shiga toxin 1, Shiga toxin 2

## Abstract

Shiga toxin-producing *Escherichia coli* (STEC) are a group of diarrheagenic bacteria associated with foodborne outbreaks. Infection with these agents may result in grave sequelae that include fatality. A large number of STEC serotypes has been identified to date. *E. coli* serotype O104:H4 is an emerging pathogen responsible for a 2011 outbreak in Europe that resulted in over 4000 infections and 50 deaths. STEC pathogenicity is highly reliant on the production of one or more Shiga toxins that can inhibit protein synthesis in host cells resulting in a cytotoxicity that may affect various organ systems. Antimicrobials are usually avoided in the treatment of STEC infections since they are believed to induce bacterial cell lysis and the release of stored toxins. Some antimicrobials have also been reported to enhance toxin synthesis and production from these organisms. Various groups have attempted alternative treatment approaches including the administration of toxin-directed antibodies, toxin-adsorbing polymers, probiotic agents and natural remedies. The utility of antibiotics in treating STEC infections has also been reconsidered in recent years with certain modalities showing promise.

Shiga toxin-producing *Escherichia coli* (STEC) are a group of bacterial organisms that are capable of producing one or more types of Shiga toxin (Stx). STEC are associated with a disease spectrum ranging from diarrhea and hemorrhagic colitis (HC) to the potentially fatal hemolytic uremic syndrome (HUS) and thrombotic thrombocytopenic purpura (TTP). STEC infections are typically food-borne (Dupont, 2007) and the production of Shiga toxins (Stx1, Stx2 or a variant) is believed to be central to the pathogenesis of these organisms. STEC strains are the result of an insertion of one of a group of lysogenic lambdoid bacteriophages that harbor an Stx1/2-encoding gene into the *E. coli* genome. The clinical syndromes, pathogenic characteristics, the pathobiology of these organisms and the toxins they produce are reviewed in Melton-Celsa et al. (2012); Farrokh et al. (2013); Kruger and Lucchesi (2015).

In recent years, novel serotypes have emerged culminating in a major outbreak in 2011 caused by a novel pathotype, *E. coli* O104:H4. The review at hand focuses on potential treatment strategies for STEC infections in light of a consensus contraindication of employing antimicrobials for these bacterial pathogens. The rise of *E. coli* O104:H4 and approaches employed in its treatment are highlighted.

## Emerging STEC serotypes

A large number of STEC serotypes has been documented; these have been isolated from various types of animals including cattle, sheep, and goats (Farrokh et al., 2013). More than 380 STEC serotypes have been associated with human disease; some of the most frequently reported serotypes include O111:H-, O26:H11/H-, O103:H2, O113:H21, O91:H21/H-, O117:H7, O118:H16, O121:H19, O145:H28, O128:H2/H-, and O146:H21. The O157:H7 serotype has been the most commonly isolated one in association with HC and HUS in both outbreaks and sporadic cases. It accounts for more than 30% of estimated STEC illness and mortality cases in the United States (Karmali et al., 2010; Scallan et al., 2011). However, there are some indications that non-O157 STEC are gaining traction in the United States and that they may be even more common than O157 strains in severe illnesses caused by STEC in parts of Europe, Latin America, Australia, and Africa (Blanco et al., 2005; Wang et al., 2013).

The epidemiology and pathogenic characteristics of non-O157 serotypes are not well studied; however, the limited reported data indicates some differences between the two types of infections. Non-O157 strains appear to induce a longer period of diarrhea which is less frequently of the hemorrhagic type (Johnson et al., 2006). Nevertheless, studies demonstrate that these non-O157 serotypes can be as virulent as O157 serotypes depending on the strain involved (Ethelberg et al., 2004).

Perhaps highlighting the relevance of monitoring these non-O157 serotypes was the emergence of the rather notorious *E. coli* O104:H4. This novel pathogen was the cause of a 2011 outbreak that affected 16 European countries with the majority of cases reported in Germany. Few cases were reported in Canada and the United States as well; nevertheless, these were travelers who had been to Europe prior to becoming ill. Reports of this novel pathogen started in May of 2011 and had peaked and then dwindled by July of the same year due to control measures that were implemented. The WHO indicates that 4075 cases and 50 deaths were caused by this STEC outbreak. Therefore, a 1.23% mortality rate was observed. On the other hand, the mortality rate of HUS due to *E. coli* O104:H4 in this outbreak was 3.74% (WHO, 2011). *E. coli* O104:H4 appears to be an enteroaggregative *E. coli* (EAEC) that has acquired the ability to produce Stx2, typically produced by enterohemorrhagic *E. coli* (EHEC) rather than EAEC group members. This may have occurred via horizontal gene transfer resulting in a new *E. coli* virotype dubbed the Enteroaggregative Hemorrhagic *E. coli* or EAHEC (Bloch et al., 2012). The O104:H4 serotype harbors two copies of the Stx2-encoding prophage. Therefore, this emergent bacterium seems to have a rather novel epidemiologic and pathogenic profile (Brzuszkiewicz et al., 2011; Mellmann et al., 2011). While ruminants are the reservoir of most STEC serotypes, no animal reservoir has been identified for *E. coli* O104:H4 and humans are believed to be the major reservoir for this organism (Wieler et al., 2011; Auvray et al., 2012; Karch et al., 2012). Whereas, the clinical profile of *E. coli* O104:H4 was relatively similar to that caused by other STEC infections some pertinent differences existed. For instance, about a quarter of subjects affected developed HUS during the 2011 outbreak, which is 2–5 fold higher than the rate usually observed for an STEC infection (WHO, 2011).

## Treatment of an STEC infection

The lack of an effective treatment strategy for an STEC infection has made these agents a prominent public health threat and a burden to the medical community at large. The currently recommended management of an STEC infection mainly relies on supportive therapy and hydration (Thorpe, 2004). The use of antimicrobial agents in treating these infections has been associated with an increased risk of HUS and is therefore contraindicated (Qadri and Kayali, 1998; Guerrant et al., 2001; Safdar et al., 2002).

## Novel and alternative STEC treatment strategies

The debatable use of antimicrobial agents for the treatment of an STEC infection has led to the rise of various alternative treatment approaches (Table 1). These have ranged from the use of natural products to the development of novel regimens that re-examine employing antimicrobials.

**Table 1:** **Experimental approaches to the treatment of Shiga toxin-producing *Escherichia coli* infections**.

**Approach**	**Method**	**Reference(s)**
Shiga toxin receptor analogs	Carbosilane dendrimers with terminal Gb3 moieties	Nishikawa et al., 2002, 2005
	Multivalent carbohydrate compounds	Kitov et al., 2000; Mulvey et al., 2003
	Gb3 polymers with highly clustered trisaccharides	Watanabe et al., 2004
	Toxin receptor mimic-producing bacteria	Paton et al., 2000, 2001; Hostetter et al., 2014
	Pk trisaccharide bound to a sorbent medium	Trachtman et al., 2003
Intracellular interference with Shiga toxins	Ac-PPP-tet	Watanabe-Takahashi et al., 2010
	TVP	Stearns-Kurosawa et al., 2011
	Manganese	Mukhopadhyay and Linstedt, 2012; Gaston et al., 2013
	Retro-1, Retro-2, Retro-2^cycl^	Stechmann et al., 2010; Noel et al., 2013
Antibodies	Anti-lipopolysaccharide antibodies	Paton et al., 1998
	Monocolonal anti-Stx A subunit antibodies	Islam and Stimson, 1990
	Bovine colostrum anti-Shiga toxin antibodies	Huppertz et al., 1999; Kuribayashi et al., 2006, 2009; Seita et al., 2013
	Humanized monoclonal anti-C5 (Eculizumab)	Lapeyraque et al., 2011; Kielstein et al., 2012; Menne et al., 2012; Delmas et al., 2014
Natural Products	Lactic acid	Pittman et al., 2012
	Fruit juices	Nogueira et al., 2003
	Plant, fruit and root products, teas or extracts	Tomita et al., 1997; Isogai et al., 1998; Okubo et al., 1998; Takahashi et al., 1999; Heredia et al., 2005; Takemasa et al., 2009; Lacombe et al., 2010; Lee and Stein, 2011; Voravuthikunchai et al., 2012; Liu et al., 2013; Pellarin et al., 2013
	Green tea extract with an antimicrobial agent (Levofloxacin)	Isogai et al., 2001
Novel/Alternate approaches using antimicrobial agents	Meropenem, chloramphenicol and fosfomycin	Corogeanu et al., 2012
	Ciprofloxacin	Corogeanu et al., 2012; Geerdes-Fenge et al., 2013
	Azithromycin	Nitschke et al., 2012; Nassar et al., 2013
	Rifampicin and Gentamicin	Kanbar et al., 2003; Matar and Rahal, 2003; Rahal et al., 2011a,b; Nassar et al., 2013; Fadlallah et al., 2015
	Imipenem	Nassar et al., 2013

### Shiga toxin receptor analog

Various agents that mimic Stx receptors and bind them thus reducing their availability to cellular receptors have been developed. Carbosilane dendrimers harboring Gb3 at their termini neutralize Shiga toxins *in vitro* and were demonstrated to protect challenged mice when administered intravenously (Nishikawa et al., 2002, 2005). Similarly, multivalent carbohydrate compounds, such as STARFISH and Daisy also neutralize Shiga toxins *in vitro* and in animals (Kitov et al., 2000; Mulvey et al., 2003). Gb3 polymers with highly clustered trisaccharides bind Shiga toxins with high affinity and protect challenged mice when administered orally (Watanabe et al., 2004). Recombinant bacterial strains that express toxin receptor mimics have also demonstrated a potential efficacy *in vitro* and upon testing in animals (Paton et al., 2000, 2001; Hostetter et al., 2014). SYNSORB Pk, a synthetic Stx receptor analog consisting of a Pk trisaccharide bound to Chromosorb® P, a multipurpose sorbent medium, was shown to have an abrogative effect on Shiga toxins *in vitro*. This agent, however, was not effective in clinical trials (Trachtman et al., 2003).

### Intracellular interference with Shiga toxins

Cell permeable agents that can bind Stx2 and potentially interfere with its intracellular trafficking have been reported. These include Ac-PPP-tet (Watanabe-Takahashi et al., 2010) and TVP (Stearns-Kurosawa et al., 2011); both agents have been tested in animal models and have displayed Stx2 neutralization abilities. Manganese has also been reported to interfere with intracellular trafficking of the B subunits of Stx and to protect against Stx1 in mice (Mukhopadhyay and Linstedt, 2012). However, it did not protect against Stx1-S or Stx2a and hence it may be of limited use (Gaston et al., 2013). The small molecule inhibitors Retro-1 and Retro-2 have also been identified via high throughput screening as agents that interfere with Stx trafficking (Stechmann et al., 2010) and a derivative of Retro-2, referred to as Retro-2^cycl^, was shown to protect cells in culture against Stx (Noel et al., 2013).

### Antibodies

Preparations of antibodies that can bind Shiga toxins and neutralize their effects have been reported. Anti-lipopolysaccharide antibodies have shown protective abilities upon laboratory assessment (Paton et al., 1998) and monocolonal anti-Stx A subunit antibodies have demonstrated potential utility in both laboratory and animal studies (Islam and Stimson, 1990). Bovine colostrum antibodies against Shiga toxins have also been demonstrated to protect challenged animals (Kuribayashi et al., 2006, 2009; Seita et al., 2013). A bovine colostrum preparation, rich in immunoglobulins and harboring a high titer of anti-Stx1 and anti-Stx2 antibodies, has also been assessed; a colostrum-treated group of 13 patients and 14 placebo-treated controls were compared. The median frequency of stool excretion was decreased in the colostrum-treated patients; however, the presence of the bacterial agent in subject stools was not notably affected. Study subjects were not monitored for the effect of this treatment on the development of HUS or other potential sequelae of infection (Huppertz et al., 1999). Eculizumab, a humanized monoclonal antibody against complement component 5 (C5), was shown in small clinical studies to have beneficial effects on recovery from STEC-associated HUS including cases during the 2011 *E. coli* O104:H4 outbreak (Lapeyraque et al., 2011; Delmas et al., 2014). However, some reports have indicated that inclusion of eculizumab in the treatment of *E. coli* O104:H4-induced HUS results in no additional benefits (Kielstein et al., 2012; Menne et al., 2012).

### Natural products

Various natural products have been considered as potential therapeutic agents for STEC infections. These have included lactic acid (Pittman et al., 2012), fruit juices (Nogueira et al., 2003) in addition to plant, fruit and root products, teas or extracts (Tomita et al., 1997; Isogai et al., 1998; Okubo et al., 1998; Takahashi et al., 1999; Heredia et al., 2005; Takemasa et al., 2009; Lacombe et al., 2010; Lee and Stein, 2011; Voravuthikunchai et al., 2012; Liu et al., 2013; Pellarin et al., 2013). These products have shown promise *in vitro* or in experimental animal models; however, they have not been evaluated in clinical studies. Worth noting is a study that showed a synergistic effect between green tea extract and an antibiotic, levofloxacin, in the treatment of an STEC-infected mouse model (Isogai et al., 2001) indicating that a potential risk imparted by an antibiotic treatment may be lessened by the inclusion of another agent.

### Antimicrobial agents

The use of antimicrobial agents in treating STEC infections has been controversial and the subject of an ongoing debate. While some studies indicated that the use of particular agents may increase the risk of HUS, others have reported a decrease of this risk upon implementation of antimicrobials. While these observations may be particular to certain agents at some doses, the potential risk of antimicrobial treatment inducing HUS has led to a general contraindication of such agents (Qadri and Kayali, 1998; Guerrant et al., 2001; Safdar et al., 2002). Antimicrobials are thought to augment the risk of HUS by enhancing the release of Shiga toxins from bacterial cells via a number of ways. DNA damage that can be caused by some antimicrobials may trigger the bacterial SOS response in STEC cells. The SOS response, whose function is to cope with genomic damage, results in the expression of a number of proteins that may activate the lytic cycle of the bacteriophage encoding a Stx thus enhancing its production. Other types of physiologic stresses caused by antimicrobial agents may also trigger the lytic cycle and result in increased toxin expression (Kimmitt et al., 2000; Los et al., 2009). On the other hand, Stx1 is known to be stored within the periplasmic space of STEC cells; therefore, cellular lysis induced by an antimicrobial agent may result in an enhanced release of this particular type of Stx (Strockbine et al., 1986; Yoh et al., 1997; Sato et al., 2003; Shimizu et al., 2009).

Several antimicrobial agents have been shown to enhance the release or the production of Shiga toxins from STEC cells *in vitro*; these include the quinolones, trimethoprim, and furazolidone (Kimmitt et al., 2000); however, observations indicate that these effect may be strain and antimicrobial agent-specific (Grif et al., 1998). For example some isolates of *E. coli* O104:H4 from the 2011 outbreak in Europe do not display an increase in toxin production upon treatment with meropenem, ciprofloxacin, chloramphenicol, or fosfomycin, unlike *E. coli* O157:H7 (Corogeanu et al., 2012). Our group assessed the effect of sub-MIC levels of various antimicrobial agents on triggering the SOS response and the production of Shiga toxins in *E. coli* O157:H7 and in *E. coli* O104:H4. A sub-MIC concentration may, after all, be the concentration available locally at the site of infection. We noted that the response is variable depending on the isolate used and the concentration of antimicrobial implemented (Nassar et al., 2013; Fadlallah et al., 2015).

Reconsideration of treating STEC infections with antimicrobial agents has nevertheless gained ground in recent years. Ciprofloxacin was recently reported to decrease the risk of HUS in subjects infected with *E. coli* O104:H4 during the 2011 outbreak (Geerdes-Fenge et al., 2013) and a reduced duration of carriage of the organism in subjects treated with azithromycin was detected during this outbreak as well (Nitschke et al., 2012). Worth noting, however, is that only a small number of treated subjects was included in both studies. Our group assessed the use of rifampicin at a concentration that decreases toxin production, but at which *E. coli* O157:H7 cells remain viable, followed by treatment with gentamicin at a bactericidal concentration. This strategy was effective in decreasing toxin release compared to solely treating the cells with a bactericidal gentamicin concentration (Kanbar et al., 2003; Matar and Rahal, 2003). Applying a similar strategy in an *E. coli* O157:H7 infection mouse model resulted in an improved animal survival rate (Rahal et al., 2011a,b). Utilizing the same strategy to treat *E. coli* O104:H4 infected mice similarly resulted in an improved survival rate compared to untreated control mice that were infected with the organism; however, the highest survival rate observed was with mice treated with gentamicin alone, unlike our observations with *E. coli* O157:H7 (Fadlallah et al., 2015). This again highlights observations indicating that different STEC serotypes and even isolates of the same serotype respond differently to antimicrobial treatments.

## Probiotics, phages and vaccines

Although probiotics may not have a therapeutic benefit in the management of an STEC infection, they may have a relevant preventative utility. Probiotics are probably capable of disrupting host-infectious agent/toxin interactions by occupying cellular receptors themselves, by producing decoy receptors that take up the toxins or by modifying the local milieu, hence making these interactions unfavorable (Corr et al., 2009). Multiple studies have shown *in vitro* beneficial effects of probiotics and that inoculation of animal models with a probiotic prior to an experimental STEC infection has preventative capabilities (Asahara et al., 2004; Reissbrodt et al., 2009; Eaton et al., 2011; Mogna et al., 2012; Chen et al., 2013; Kakisu et al., 2013; Rund et al., 2013; Stanford et al., 2014). The extent of probiotic protective capabilities seen in experimental models is likely dependent on the probiotic strain used and its ability to modify the surrounding medium. For example, the production of acetate by the probiotic agent has been demonstrated to be an important factor (Fukuda et al., 2011, 2012) and the production of butyric acid and lactic acid may be of relevance as well (Ogawa et al., 2001; Takahashi et al., 2004). One *in vitro* study showed that cultivation of STEC organisms in the presence of various *Bifidobacterium*, *Pediococcus*, and *Lactobacillus* strains results in a decreased production of Stx2. This was attributed to a decrease in pH due to the acids produced by these agents (Carey et al., 2008). Worth noting is the recombinant probiotic agent that can produce toxin receptor mimics described in section 3.a. (Paton et al., 2000, 2001; Hostetter et al., 2014). Also of relevance are the various studies indicating that the administration of probiotic agents to cattle may reduce their carriage of STEC organisms (systematically reviewed in Sargeant et al., 2007), hence effectively reducing the risk of transmitting these toxigenic agents.

Another preventative measure proposed as a means of controlling STEC is the application of lytic phages. Lytic phages have been shown to reduce STEC numbers *in vitro* (Niu et al., 2009; Rivas et al., 2010), in phage-treated food products (Abuladze et al., 2008; Anany et al., 2011), on hard surfaces (Abuladze et al., 2008), in mice and in some ruminants (Raya et al., 2006; Sheng et al., 2006). Phage-containing products that can be sprayed on animal hides or on meat products for the control of STEC organisms are available on the market and are Food and Drug Administration (FDA) approved (Sillankorva et al., 2012). The efficacy of orally treating cattle with lytic phages, however, was reported to be limited and requires the development of an enhanced approach or delivery mode (Stanford et al., 2010). Bacteriophages used to eradicate STEC agents may also have a therapeutic utility should the safety and efficacy of such an application be demonstrated in humans.

Various vaccine approaches have also been attempted including the development of preparations that contain bacterial peptides and virulence factors (Wen et al., 2006; Tiels et al., 2008; Gu et al., 2009; McNeilly et al., 2010; Asper et al., 2011; Cai et al., 2011; Gupta et al., 2011; Wan et al., 2011; Zhang et al., 2012; Rossi et al., 2013; Sato et al., 2013; Cernicchiaro et al., 2014; Garcia-Angulo et al., 2014; Lu et al., 2014; Mejias et al., 2014; Paddock et al., 2014), attenuated bacterial cells (Rojas et al., 2010; Gu et al., 2011; Fujii et al., 2012), bacterial envelope/membrane derivatives (Cai et al., 2010; Choi et al., 2014) in addition to DNA vaccines (Bentancor et al., 2009; Ren et al., 2013). These vaccine preparations have been assessed in animal models with some showing promising results (reviewed in Garcia-Angulo et al., 2013).

In conclusion, despite the passage of more than three decades since STEC organisms were first associated with human clinical illness (CDC, 1982), a generally-accepted successful therapeutic method for these organisms remains undocumented. Various approaches have nevertheless been attempted including ones that reconsider the implementation of antimicrobial agents; beneficial effects have been reported for some agents with outcomes appearing dependent on the antimicrobials used, their dose and the STEC isolate itself. Further studies examining antimicrobial agents in the therapy of STEC infections should be conducted in animals to select the safest and most efficacious regimen that would then be assessed in clinical trials.

### Conflict of interest statement

The authors declare that the research was conducted in the absence of any commercial or financial relationships that could be construed as a potential conflict of interest.
